# Parental Educational Needs During the NICU Stay: Mothers’ Perspectives

**DOI:** 10.3390/children13010126

**Published:** 2026-01-14

**Authors:** Welma Lubbe, Kirsten A. Donald

**Affiliations:** 1 Division of Developmental Paediatrics, Department Paediatrics & Child Health, Red Cross War Memorial Children’s Hospital, Neuroscience Institute, University of Cape Town, Private Bag X3, Rondebosch 7701, South Africa; kirsty.donald@uct.ac.za; 2 NuMIQ Research Focus Area, Faculty of Health Sciences, North-West University, Private Bag X6001, Potchefstroom 2520, South Africa

**Keywords:** neurodevelopmental care, premature infant, parent education interventions, parent education programmes, parent perceptions

## Abstract

**Highlights:**

**What are the main findings?**

- Mothers identified key educational needs regarding basic infant care, infant health/behaviour, and post-discharge preparation.
- Preferred programme features included visual materials, verbal instruction with demonstrations, group discussions, and consistent, knowledgeable facilitators.

**What are the implications of the main findings?**

- Parent education programmes should be context-specific and aligned with mothers’ real-time NICU experiences.
- Addressing psychological, communication, and practical support needs is essential for parents to fully engage in and benefit from education.

**Abstract:**

Background: Parents caring for preterm infants during hospital admission have unique needs. How these are addressed plays an important role in parents’ ability to cope with caregiving responsibilities. Educational programmes have proven beneficial to parents during their infant’s stay in the neonatal intensive care unit (NICU), for both parental and neonatal outcomes. Key components of parenting education during the NICU stay have been described; however, less is known about our understanding of parents’ educational needs, specifically in the South African context. Objectives: To explore parental needs and perceptions regarding a parenting education intervention provided to them while in the NICU, with a focus on programme content, structure, and mode of delivery. Methods: Three focus group discussions were conducted with mothers of preterm infants admitted to the NICU of a referral hospital in the North West province, South Africa. Inclusion criteria comprised parents of infants born in the hospital, singletons or multiples, with a gestational age below 37 weeks, and expected to stay in the NICU for at least 7 days. Discussions centred on mothers’ perceived needs regarding parenting education based on their experiences during their baby’s NICU admission. Results: Twenty-five mothers of singletons or multiples born before 37 weeks of gestation participated in the study. Three main themes were identified: (1) preference for content topics to include basic infant care, infant health and behaviours, and post-discharge related information; (2) education programme structure, which included instructional approaches and training logistics; and (3) support needs, including intrapersonal motivators, communication, and psychosocial and physical support. Conclusions: Participants recognised educational content needs that align with existing literature. However, they also emphasised the importance of addressing basic physical and emotional needs while receiving educational content, ensuring that parents feel empowered and capable of engaging with the information.

## 1. Background

The complexities associated with preterm birth are often traumatic for parents, both physically and emotionally [1]. Parents experience separation from their infants in the neonatal care environment, while strangers’ care for their preterm babies’ feelings of being poorly prepared, overwhelmed by both in-hospital and post-discharge caretaking responsibilities, and unique personal needs throughout an infant’s stay in the neonatal intensive care unit (NICU) may influence parents’ ability to cope with their responsibilities. These factors may have an impact on the development of the parent–infant relationship as well as the caregiver’s experience of their parenting role [2].

Educational programmes delivered to parents of preterm infants during their NICU stay have been shown to improve both parental and neonatal outcomes [3]. These benefits include increased parental confidence, enhanced knowledge of infant care, greater parental satisfaction, and improved mental health, such as reduced stress and anxiety [3,4]. These programmes have the potential to alleviate parental stress and contribute to discharge preparation [4,5]. Springer et al. [4] confirmed previously identified outcomes, including enhanced caretaking behaviour, positive parent–infant interaction, and stronger attachment. Additionally, Melnyk et al. [6] demonstrated a reduction in the length of stay when parents participated in NICU-based educational programmes.

Drawing on previous studies, educational topics included in preterm parenting educational programmes covered infant health, information about what to expect in the NICU, preparing parents to see their infant for the first time, understanding infant behaviour, practical aspects of infant care (including skin-to-skin care and infant massage), infant feeding with a high emphasis on breastfeeding, and discharge planning. In addition, Brett et al. [5] identified potentially helpful education interventions, such as teaching parents to use behavioural assessment scales to interpret their preterm infants’ behaviour, parent support forums, and information and communication sharing.

There is evident diversity of focus in studies on parent education programmes [3], with most evaluating the effectiveness of education programmes. There has also been some evaluation of educational programmes with different styles of delivery, including direct instruction, written instruction (which may include brochures and reflective journaling), and even technology-based or simulation formats [4]. However, the detailed curriculum or key components to be included in such educational programmes are less frequently presented in published articles.

In a review of the current literature, Lubbe et al. [7] identified the key components of parenting education programmes for parents of preterm infants while in the NICU. The main topics included in published educational programmes were understanding the NICU environment, infant health, behaviour, care, feeding, and discharge preparation. It was clear that, for most programmes described, logistical considerations informed the delivery framework, and this informed delivery method(s), characteristics, and presentation formats of the educational intervention, and the key people involved in the educational programme delivery. The geographical representation of reports where parental educational programmes were implemented included high-, upper-middle-, and lower-middle-income countries, with one report from a low-income country. Only a few of the articles included qualitative research [7].

Although the literature covered parent educational programmes and their content designed for parents from all income levels, it did not specifically explore the South African context and parents’ perspectives of their own educational needs while their child was in the NICU. The literature therefore presents a good general framework of the ‘what’ to be included in a parenting programme, but is less clear about the ‘when’, ‘by whom’, and ‘how’ questions. It remains important to explore the specific context of any such programme, in order to determine the most suitable structure, considering both instructional approaches (such as delivery method and instructional formats) and training logistics (frequency and time of educational sessions and facilitator type).

The aim of this study was to explore parental needs and perceptions regarding a parenting education intervention provided to them while in the NICU, with a focus on programme content, structure, and mode of delivery.

## 2. Methods

### 2.1. Aim and Design

This study followed a qualitative, exploratory, and descriptive design comprising three semi-structured focus group discussions (FGDs) conducted between February and April 2023 with parents of preterm infants admitted to the neonatal ward.

### 2.2. Setting

This study was conducted in the Dr Kenneth Kaunda district in the North West province of South Africa, in a public, tertiary, referral hospital. The hospital has eight NICU beds, nine high-care neonatal beds, and 28 low-care neonatal beds, with an average of 66 neonatal admissions per month [8]. The term neonatal intensive care unit (NICU) in this study included critical care, high care, growing prem, and kangaroo-mother care units, as these are all included in a neonatal ward context in South Africa.

The patient profile of this hospital includes mothers from lower-income settings without medical insurance, who are often referred to the facility from other geographical districts. Immediately after delivery, mothers are admitted to the postnatal ward, and mothers of less stable or more immature infants are discharged and may visit their babies daily (or less often) from home. Parents often live some distance from the hospital and incur costs when visiting their infants. Lodging facilities are available only when babies are mature enough to be cared for in the kangaroo-mother unit.

### 2.3. Participants

Inclusion criteria comprised parents of infants, singletons or multiples, born in the hospital with a gestational age below 37 weeks, and expected to stay in the NICU for at least 7 days. Mothers aged over 18 years were enrolled during their infants’ hospital stay as soon as possible after admission to the NICU. Parents needed to understand and speak either English or one of the regional languages (Setswana or Afrikaans). The most frequently spoken home language in the province is Setswana (63.4%), followed by Afrikaans (9%). Parents with a history of drug addiction, psychosis, or other severe mental illnesses were excluded. Parents whose infants had severe congenital abnormalities or who had undergone major surgery were also excluded, due to the very specific educational needs surrounding these conditions.

Parents meeting specific inclusion and exclusion criteria were purposefully selected [9], as these mothers were able to provide in-depth information on the topic of parental educational needs while their infant was admitted to the NICU. The mothers were allocated to three face-to-face focus groups consisting of eight to nine participants each [10], totalling 25 mothers.

### 2.4. Recruitment of Participants

The study received ethical approval from the Health Research Ethics Committee of the University of Cape Town (UCT-647/2021), followed by North West provincial and hospital management approvals. No data collection procedures were initiated prior to obtaining written informed consent from all participants. This study adhered to the principles of the Declaration of Helsinki [11] and the 2024 South African Ethics in Health Research Guidelines [12].

Mothers who met the inclusion criteria were recruited by dedicated nursing staff working in the neonatal ward. Recruitment nurses explained the study to the mothers and invited them to attend a focus group in a quiet and private venue on the hospital premises at a convenient time that would not interfere with feeding or other contact times with their infants.

Parents who wished to consider their participation in the study or consult with family members were encouraged to decide within 24 h, since the information to be obtained was time-sensitive and mothers’ input was required while they were living the experience of having their baby admitted to the NICU. The first FGD was conducted after the first 10 mothers were enrolled, and the process was repeated for the second and third focus groups.

At the venue, the researcher (lead author) explained the study again, after which the researcher left the room and allowed an independent person to conduct the informed consent process, once again explaining that the mothers could opt out without any prejudice to themselves. Some mothers did, indeed, choose to withdraw at this stage (that is, prior to consent). Permission was also obtained to audio-record the discussions to ensure that no data were missed.

### 2.5. Data Collection

FGDs provided the opportunity to interview a homogeneous group of mothers—all with preterm infants still admitted to the NICU—on a specific topic: their perceived educational needs [13]. The strength of focus group interviews in this study was the ability to capitalise on the group process—with interaction and participants sharing experiences as well as commenting on each other’s experiences, producing more detailed information and a deeper perspective [14] of their own educational needs while in the NICU.

A focus group discussion question guide was developed for the purpose of the study (Supplementary Material S1). The main question which guided the FGDs was “What are/were your needs regarding parenting education while your baby is/was admitted to the NICU?” In addition, probing questions guided parents to share specific content to be included in a parenting education programme, share their ideas on the mode of delivery and structure of the delivered programme, reflect on how healthcare professionals influenced their parenting, and share any creative solutions they had regarding NICU-based parenting education.

The FGDs lasted between 36 and 58 min (an average of 46 min) and were conducted by an experienced interviewer with the support of a research intern who took field notes. Saturation was reached within each FGD as well as between the three groups [15].

### 2.6. Data Analysis

The audio-recorded interviews were transcribed verbatim by a trained transcriber and checked for accuracy. Setswana transcripts or phrases were translated into English by a language practitioner to allow English-speaking researchers to analyse the data. To ensure anonymity, a code was allocated to each focus group [16], and all identifiers were removed. Thematic analysis of the FGDs and field notes was performed by the researcher and an independent coder according to Tesch’s approach [17]. This process was followed by reading and re-reading all the transcripts and field notes to obtain a sense of the whole, whereafter one focus group was selected and the salient categories were identified. Similar topics were identified and clustered together, while appropriate and descriptive wordings were identified to assign the topics to appropriate sections. The next step was to group related topics and categorise them, whereafter final categories were agreed upon, which were then organised and coded. This structured data was preliminarily analysed and re-coded where necessary.

**Figure 1:**
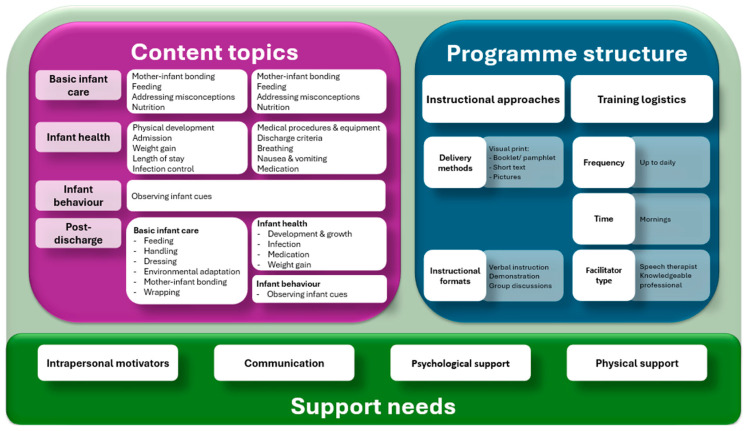
Parent educational programme delivery framework.

All anonymised copies of the transcripts were sent to the co-coder, who replicated the process to ensure that no data were lost. The coder and co-coder then compared the analysed datasets to identify, discuss, and reach a consensus on the discrepancies in the final codes, categories, and themes [16]. This process and results can be seen in Figure 1: Parent educational programme delivery framework.

## 3. Results

Participating mothers were over 18 years of age and had experienced either a singleton or multiple birth, with a reported infant birth weight ranging between 800 and 1900 g. Most mothers were fluent in English, with a small percentage preferring to speak Setswana but understanding English. Biographical data was requested from the participants in a self-reported format as part of their informed consent. However, most participants were unsure about information such as exact gestational age, birth weight, and APGAR scores. Since ethical approval did not allow access to patient files, the researchers relied on information that the mothers shared during the focus group interviews.

The findings are presented in three main areas: (a) content topics, (b) education programme structure, and (c) support needs, aligning well with the questions posed, namely the following: (1) ‘share specific content’, (2) ‘share ideas on structure’, and (3) ‘mode of delivery’ (see Figure 1: Parent educational programme delivery framework). Participants were also asked to reflect on how healthcare professionals influenced their parenting and to provide creative ideas regarding parenting education.

The results are now presented under the three categories: **Content topics**, **Programme structure**, and **Support needs**.

### 3.1. Category A: Content Topics

Content topics included information needs about basic infant care, infant health and behaviours, and post-discharge related information needs.

Basic infant care needs voiced by parents focused on information pertaining to feeding (*n* = 42), including information on breastmilk expression, bottle-feeding versus breastfeeding, and milk storage. As participants stated, ‘Because they also told us, before you feed you start to check if the tube is on the right position’ (F1) and, ‘Yes, we want more information about feeding and bathing’ (F3). Basic infant care further included handling (*n* = 14) as voiced by F1: ‘So, as parents we are told to not touch our babies because we bring infection’. One mother’s response, which highlighted the importance of their innate need for mothering and closeness, was, ‘We take time to come to the babies and bond with them’ (F1).

Infant health and infant behaviour focused mostly on concerns regarding infant development (*n* = 20), as articulated by one participant, ‘I need to know that he is developing … like lungs, and others, after how long.’ (F1). The next most prominent focus was on medical procedures or equipment (*n* = 12), ‘Mentally wise, it won’t affect them because, most of them, you find out that they’re putting drips on their heads?’ (F1). Infection was mentioned (*n* = 9), ‘Like it is said they are prone to infection’ (F1), followed by weight gain (*n* = 7) as an indicator of good development and as a discharge criterion, ‘When he reaches that KG [kilogram] where he can go home’ (F1). Since breathing is a medical challenge evident in all preterm infants, it was not surprising that information on it was specifically voiced as an educational need by parents (*n* = 9) and was formulated by a mother from F2 as, ‘If something happens to the infant; they don’t breathe properly or forget to breathe? If he forgets to breathe, what should I do?’. Discharge criteria were mentioned least often (*n* = 7), which can be attributed to mothers being at an early stage in their NICU journey, with their baby not yet ready to go home. Mothers did, however, ask questions such as, ‘When is he supposed to be discharged?’ (F2) and ‘Is the baby stable to go home?’ (F3), supporting the view that discharge planning should be initiated early during the hospital stay.

Infant behaviour was the third-most frequently raised issue (*n* = 24). It became clear that some mothers were tuned-in to infant behaviour, with one observing, ‘They’re stressing too much’ (F1), and another, ‘Sometimes you feel that your child is not right’ (F2). Another pondered, ‘Is the baby feeling well or whatever?’ (F3). Mothers did not necessarily seem to have the language to describe infant behaviour, but they did think about it and wanted to know more.

Post-discharge related information. Although exploring educational needs during the NICU stay was the focus of the FGDs, it is important to note that mothers were already thinking about what their needs would be after discharge. These were similar to their perceived in-hospital needs, with feeding highlighted as the most important (*n* = 30), evident from a question: ‘How long after discharge must I continue breastfeeding?’ (F1). Feeding is the most important mechanism in parent–infant interaction and is directly linked to bonding and maternal emotional health; therefore, it was expected to be the highest education need as well.

Education about infant development (*n* = 20) remained high in the NICU and after discharge, with information sought on handling (*n* = 8) decreasing but still evident as a post-discharge need. This may indicate that mothers already learn/learnt this information and skills during their infants’ NICU stay.

### 3.2. Category B: Programme Structure

The second main theme relates to the suggested structure of a parent education programme, especially in the South African public sector context, from parents’ perspectives. In this context, ‘structure’ refers to instructional approaches and training logistics.

Instructional approaches included visual format delivery methods. Participants proposed a training booklet (*n* = 5) or pamphlet (*n* = 3), with short descriptions (*n* = 2) and pictures (*n* = 3) explaining the necessary information. Mothers stated, ‘The information booklet requires comprehensive information on the development of the baby’ (F3), They [mothers] read the first page and then they’re tired’(F1), and ‘It’s fine with pictures’ (F1). Additionally, participants suggested instructional formats such as verbal instruction (*n* = 20), augmented by demonstrations (*n* = 12), and group discussions (*n* = 11). A participant stated, ‘I think with the discussion, it will help because some of the mommies they are lazy to read’ (F1). The programme presenter should be knowledgeable and be either a speech therapist (*n* = 3)—since, ‘You must see the speech therapist’ (F2)—or a qualified doctor (*n* = 2), rather than an intern, since ‘Qualified doctors have more information than interns’ (F3).

Feedback on training included the duration, timing, frequency, and facilitator type for the educational intervention. Participants mentioned that the frequency of sessions should include continuous learning opportunities (*n* = 2) that occur ‘Almost every day’ (*n* = 6) and preferably occur in the mornings (*n* = 8), since ‘We came here at six o’clock’ (F1). The duration of sessions was not mentioned by mothers; however, it was clear that such sessions should fit in between feeding times and not be longer than 45 min, based on mothers’ availability to participate in the FGD and tolerance for being away from their infants.

### 3.3. Category C: Support Needs

Support seemed to influence maternal experiences either positively or negatively, and if mothers’ support needs were not addressed, they seemed unable to identify their own learning needs. Support can be sub-categorised as intrapersonal motivators, communication, and psychological or physical support.

Intrapersonal motivators affecting maternal experiences included perceived maternal involvement in infant care (*n* = 12) and maternal background (*n* = 9). Mothers felt unprepared to care for a preterm infant (*n* = 26), with one mother commenting, ‘Most of the mommies, when we do have these premature babies, we don’t have a heart for them’ (F1). They voiced feelings of stress (*n* = 6) and being judged or blamed (*n* = 6). A range of coping strategies were also identified, including patience (*n* = 4), self-talk (*n* = 4), and accepting the unexpected (*n* = 3).

Comments on communication highlighted negative communication and a lack of communication, especially nurse–caregiver communication (*n* = 24). Mothers mentioned negative communication, ‘Some of the nurses also they become rude, forgetting that you are not there, you didn’t choose to be there, you didn’t choose to have the child at the early’ (F1), and ‘If something bad happens, you’re the wrong one’ (F3). Lack of communication was also identified by mothers as challenging, ‘They do not let us know about the weights; they don’t let us know a lot of things’ (F1); ‘What are we supposed to do, they don’t teach us’ (F2); and ‘You were not given a chance to ask, you were not told anything, you were not updated’ (F3).

Psychological support (*n* = 8) required was evident from mothers who identified their own need for counselling; for example, ‘You need someone to refer you straight to the person who you know will help you with your problems’ (F2). Specific mental support needs were voiced as, ‘I feel most mothers are burdened but they can’t talk about what is happening inside them’ (F2). Finally, a mother from F1 sounded rather desperate when she responded with, ‘Now, I don’t know what, where or how is she. I just see her [the nurse] feeding her, changing nappy, I don’t know anything about my baby’. Peer support is an important need, as expressed by a mother stating, ‘I get healed inside and especially when I meet these people. These people, someone, can come with this advice, you took advice’ (F1).

Physical support needs included resources required, such as accommodation (*n* = 9), internet access (*n* = 4), transport (*n* = 3), financial assistance (*n* = 3), and needing antenatal information (*n* = 3). Mothers asked, ‘Why can’t they allow us to sleep here, all of us?’ and ‘You will need an extra [internet access] data for that’ (F1) and ‘I don’t have money’ (F2).

## 4. Discussion

This study identified a clear and consistent need among mothers of preterm infants for structured parenting education during the NICU stay, with educational needs clustering around three interrelated domains: (1) content topics, (2) programme structure, and (3) support needs. Mothers prioritised education on basic infant care, infant health, and behaviour, and post-discharge preparation, while simultaneously emphasising that the effectiveness of education is contingent on emotional, communicative, and practical support being in place. Importantly, mothers expressed a strong preference for education that is individualised, delivered by knowledgeable and consistent healthcare professionals, and aligned with their infant’s clinical condition and stage of NICU admission.

Taken together, the findings indicate that the central outcome of this study is not merely the identification of educational topics, but rather the need for parenting education in the NICU to be individualised. Mothers’ educational needs varied according to multiple contextual and infant-related factors, including the stage of NICU admission, perceived infant stability, anticipated length of stay, and the underlying clinical reason for admission. Content preferences, readiness to engage in education, and preferred modes of delivery were therefore not uniform, but shifted in response to these factors.

Our study is one of the very few qualitative studies exploring parental educational needs from mothers’ personal perspectives, while their preterm babies are still admitted to the NICU. Internationally, studies have focused more on evaluating the effectiveness of parent educational interventions, with less published on the process of developing educational interventions or describing the actual parent educational interventions [3,4].

In our study, the content topics category aligned well with published research, suggesting the inclusion of topics in a parenting education programme to cover basic infant care, infant health, infant behaviour, and post-discharge information.

Although the topics may be similar, further exploration is required to understand the contextual differences within each topic. For example, the visuals used to explain the NICU environment may differ to reflect each setting, and there may be differences in policy and procedures. However, both our participants and the literature confirmed the necessity of including these topics in an educational programme [18,19,20,21,22,23,24,25,26,27,28,29,30,31,32,33]. In addition, pictures explaining infant behaviour may be included to reflect the diversity and cultural background of the parents who receive the education, but infant behaviour cues and parent–infant interaction strategies are likely to remain constant across settings. Our findings align with the literature regarding infant behaviour, including infant cues, communication, and interaction, in parent educational materials [18,20,21,22,23,24,25,26,27,28,29,30,34,35,36,37,38,39,40,41].

Our study identified variations in the suggested instructional approaches and training logistics for educational interventions (education programme structure). Our participants favoured materials presented in a visual, printed format, such as a booklet or pamphlet with short descriptions and pictures, similar to the findings of Bracht et al. [19], Chen et al. [20], Heo and Oh [22], Khanjari et al. [42], Milgrom et al. [37], Morey and Gregory [43], Nieves et al. [38], Ong et al. [29], Petteys and Adoumie [39], Peyrovi et al. [30], Yu et al. [41], and Gök and Efe [44]. However, the mothers also communicated the need for verbal instructions augmented by demonstrations and group discussions. These modes fit well with the Family Integrated Care (FICare) approach, which utilises bedside demonstrations [19,45] and group discussions that also form part of Creating Opportunities for Parent Empowerment (COPE) [25], FICare [19,45], and family-centred care (FCC) [46]. Verbal instructions seem to favour individual bedside discussions, as described in the literature [19,24,26,38,47]. FICare includes individual bedside discussions and group discussions with written materials [19,45], while COPE also considers individual discussions [25] and digital formats to deliver content [38]. Gök and Efe [44] additionally reported on a web-based education programme in Turkey. In our study’s setting, mothers suggested the use of technology, such as utilising the MomsConnect messaging system, but identified challenges with online training, such as limited data and poor internet connectivity in some areas of South Africa. Our participants preferred presentations in a verbal format and preferably in groups, which may speak to the support obtained from a peer group, as well as the strong social bonds evident in African culture.

Regarding programme delivery, parents in our study suggested numerous educational sessions, even daily, and expressed a preference for mornings. We did not identify literature reporting parents’ preferences regarding the time of day for the sessions. Practical considerations mentioned by our participants around the duration of sessions included their preference for educational sessions to be fitted into the timeframe between feedings, to allow mothers to have maximum time with their babies. Our participants did not comment on a preferred length of sessions, while the consulted literature suggested that the duration varied between 15 min [47] and 120 min [21], with most studies reporting sessions of approximately 60 min to have been the most practical [18,23,26,28,30,36,44]. Therefore, a maximum length of 60 min was suggested as a practical window in our setting.

Interestingly, the conclusions of a high proportion of the reviewed literature were not consistent with our findings and suggested that nursing staff are the most common presenters of parent education programmes in low-income settings such as South Korea [22]; low–middle income countries, including Iran [23,27,28,42]; upper middle-income countries, including Brazil [33] and Malaysia [29]; and high-income countries such as China [20,41,47], the USA [38,43], Australia [45], and Canada [19]. The use of a speech therapist, also mentioned by our participants, was suggested by only one study in the USA [43]. Mothers in our study were quite emphatic that presentations should be made by knowledgeable healthcare professionals and not rotating professionals, such as students or medical interns. This highlights the opportunity to explore how nurses’ current workload may impact the available time for parental education and support. This suggests the need to enhance healthcare professionals’ training to better equip them for the NICU environment and support mothers, with further research potentially improving preparedness and outcomes.

Taken together, the findings of this study highlight the importance of including views of end-users (i.e., parents in the NICU) when designing a parent education programme for a specific setting. The views on content topics seem to be aligned across high- and low-resource settings, but practical considerations such as where, when, how, and by whom should be contextually considered.

Educational interventions do not typically serve psychological needs; however, support needs seem to influence maternal experiences, either positively or negatively. Our study highlighted the importance of support in the NICU as a building block for parents to move towards the identification of their educational needs, since mothers seemed unable to identify the latter when their support needs were not met.

Established parenting programmes such as FICare, implemented in Canada [19], Australia [45], and Spain [26], FCC, implemented in China [46] and India [24], and COPE, which originated in and was implemented in the USA [48] and proved to be effective in Iran [25], highlighted the link between NICU parental education programmes and a reduction in negative parental psychological consequences [3,49,50,51], such as parental stress and anxiety [3,19,52]. This link may therefore explain why our participants focused so strongly on their preference to have their support needs met before reflecting on their educational needs.

Our study highlights the importance of positive parental support and mothers’ traumatic experiences in the absence of respectful care. Since nursing staff are the main professional care providers, a huge responsibility lies on their shoulders to interact with parents, be available to answer questions, provide comfort during maternal–infant interaction, practice patience, and treat all parents equally. Our study suggests that resource restrictions lead to staff unavailability for this support function, as they must triage their time towards direct infant care. This results in a disconnect between parental educational needs and the available resources to address these needs. These findings suggest that effective NICU parent education should be conceptualised as a flexible, needs-responsive process rather than a fixed curriculum.

### 4.1. Limitations and Strengths of the Study

The context of our qualitative study is important, as it reflects a broader, representative setting of public health NICU contexts in South Africa, where similar challenges and dynamics are often observed. However, it is important to note that our findings are limited to a single institution, and the replication of this study in different settings would further enrich the understanding of these issues. Additionally, a limitation of our study is the language barrier, as English was not the first language of any of the participants. Although the questions were translated into Setswana by a native Setswana-speaking interviewer and the participants were encouraged to respond in their native language, there remains the possibility that some deeper meanings or nuances may not have been fully captured by the researchers. Therefore, the findings should be interpreted with caution, considering the potential impact of language-related factors.

An important limitation of this study is that detailed clinical information regarding the underlying indications for NICU admission were not available. Although all infants were born preterm and expected to remain in the NICU for at least seven days, ethical approval did not permit access to medical records, and mothers were often uncertain about specific diagnoses or clinical details. As a result, it was not possible to analyse how educational needs differed according to underlying conditions such as respiratory distress, feeding immaturity, infection, or neurological risk. Given that the indication for NICU admission may strongly influence both parental stress and informational needs, future studies should include clinical data to allow for a more nuanced understanding of how education can be tailored according to infants’ diagnoses and clinical trajectories.

Data saturation was, however, reached within and between FGDs, contributing to the credibility of the data. Credibility was further increased through member checking to ensure that the findings were accurate and reflective of the mothers’ experiences [53], and therefore, truthfully represented the population of this study [54]. The use of a skilled coder and co-coder, in conjunction with a consensus meeting, contributed to the validity of the analysis, as it ensured that multiple perspectives were incorporated into the coding process. Biases were reduced in this manner, while the consistency and reliability of the findings were enhanced.

Transferability was achieved by providing in-depth descriptions, which enables the reader to assess the relevance and applicability of the findings to a larger population [54]. Confirmability refers to the degree to which the findings of a study, particularly in qualitative research, are based on the data collected from participants and not influenced by the researcher’s biases or interpretations [54]. The authors have outlined the steps taken to ensure the integrity of the data and eliminate bias in the Methods section (above).

Although we intended to explore broader parental needs, no fathers were identified in the NICU during the study. This seemed to be the case in other settings as well, since some studies were designed with a specific focus on fathers’ roles and experiences [20]. Therefore, fathers’ needs and involvement should be explored in future studies. A limitation of our study is that biographical data were collected through participant self-reports, and we did not anticipate that mothers would not be sure of data such as birth weight and gestational age.

### 4.2. Implications for Practice and Further Research

Mothers of preterm infants may greatly benefit from a parent education programme during their NICU stay, due to its positive impact on parental and neonatal outcomes. Our study confirmed that the topics parents believe should be included in such a programme seem to be similar across focus groups, irrespective of context; however, further research should be directed towards exploring the best programme structure for various settings. A larger and more diverse sample will also allow for exploring differences in educational needs, which may exist in mothers of infants with different gestational age and birth weight, and different groups of mothers, for example, teenage mothers versus older mothers, and singleton opposed to mothers of multiples. Therefore, the development of a contextualised parent education programme for the NICU and testing its efficacy and acceptability is suggested for future research. A more in-depth evaluation of neonatal and parental outcomes when a parenting education programme is implemented as an intervention will provide valuable suggestions towards integrating this type of intervention into standard neonatal care. Peer support was briefly mentioned by the participants and could be investigated as a potential intervention to address the support component of a parent education intervention.

## 5. Conclusions

This qualitative study identified three key considerations for planning parenting education programmes in the NICU: educational content, programme structure, and maternal support needs. Mothers emphasised that education should be flexible and contextualised to their individual circumstances, and that effective education depends not only on information provision but also on respectful communication and adequate psychological and physical support. The absence of detailed information on the underlying clinical indications for NICU admission limits insight into how educational needs may differ by diagnosis, highlighting the need for future studies to incorporate clinical data when developing individualised education programmes. 

## Data Availability

The datasets generated and analysed during the study are not publicly available, in order to protect the privacy and confidentiality of the participants due to the qualitative nature of this data. Data supporting the findings are available from the corresponding author on reasonable request.

## Supplementary Materials

The following supporting information can be downloaded at: https://www.mdpi.com/article/10.3390/children13010126/s1.

## Abbreviations

COPE	Creating Opportunities for Parent Empowerment
FCC	Family-centred Care
FICare	Family Integrated Care
FGDs	Focus Group Discussions
NICU	Neonatal Intensive Care Unit
