# Supplementary material for: Parental Educational Needs During the NICU Stay: Mothers’ Perspectives
Source: Children (Basel). 2026 Jan 14;13(1):126. doi: 10.3390/children13010126 (PMC12840342; doi:10.3390/children13010126)
Supplement: Supplementary file 1 [file children-13-00126-s001.zip › children-4021021-supplementary.pdf]

Supplementary Material 1

Focus Group Discussion Question Guide

Main question

What are/were your needs regarding parenting education while your baby is/was admitted in the NICU?

Probing questions

1. What do you consider important to be included in a parenting education intervention programme while your baby is admitted to the NICU?
2. How, in your experience, did the healthcare professionals in the NICU influence your parenting in the NICU?
3. Give more details on content, mode of delivery, structure of delivery for a parenting education programme.
4. Do you have any creative solutions to share, in order to address the potential challenges?
5. Would you like to share anything else?
